# A Systematic Review of Interleukins as Diagnostic and Prognostic Biomarkers for Peripheral Artery Disease

**DOI:** 10.3390/biom13111640

**Published:** 2023-11-12

**Authors:** Niousha Djahanpour, Naiyara Ahsan, Ben Li, Hamzah Khan, Kim Connelly, Howard Leong-Poi, Mohammad Qadura

**Affiliations:** 1 Division of Vascular Surgery, St. Michael’s Hospital, Unity Health Toronto, University of Toronto, Toronto, ON M5B 1W8, Canada; 2 Keenan Research Center for Biomedical Science, Li Ka Shing Knowledge Institute, St. Michael’s Hospital, Unity Health Toronto, University of Toronto, Toronto, ON M5B 1W8, Canada; 3 Division of Cardiology, St. Michael’s Hospital, Unity Health Toronto, University of Toronto, Toronto, ON M5B 1W8, Canada; 4 Department of Surgery, University of Toronto, Toronto, ON M5B 1W8, Canada

**Keywords:** interleukin, biomarker, prediction, diagnosis, prognosis, peripheral artery disease

## Abstract

**Background:** Peripheral artery disease (PAD) involves atherosclerosis of the lower extremity arteries and is a major contributor to limb loss and death worldwide. Several studies have demonstrated that interleukins (ILs) play an important role in the development and progression of PAD; however, a comprehensive literature review has not been performed. **Methods:** A systematic review was conducted and reported according to PRISMA guidelines. MEDLINE was searched from inception to 5 December 2022, and all studies assessing the association between ILs and PAD were included. **Results:** We included 17 studies from a pool of 771 unique articles. Five pro-inflammatory ILs (IL-1β, IL-2, IL-5, IL-6, and IL-8) and one pro-atherogenic IL (IL-12) were positively correlated with PAD diagnosis and progression. In contrast, two anti-inflammatory ILs (IL-4 and IL-10) were protective against PAD diagnosis and adverse limb events. Specifically, IL-6 and IL-8 were the most strongly associated with PAD and can act as potential disease biomarkers to support the identification and treatment of PAD. **Conclusions:** Ongoing work to identify and validate diagnostic/prognostic inflammatory biomarkers for PAD has the potential to assist clinicians in identifying high-risk patients for further evaluation and management which could reduce the risk of adverse cardiovascular and limb events.

## 1. Introduction

Peripheral artery disease (PAD) is a debilitating atherosclerotic disease of the lower extremities and can be an indication of systemic atherosclerosis [1]. PAD is often misdiagnosed or left untreated as patients are asymptomatic in the early stages of disease. The hardening and subsequent narrowing of the arteries due to the buildup of atherosclerotic plaque in the lower extremities eventually leads to muscle hypoxia and can manifest as debilitating limb pain and muscle cramping during activity, known as intermittent claudication [2]. In severe cases, PAD can lead to chronic limb threatening ischemia which is associated with continuous pain at rest, night pain, and tissue loss, requiring surgical revascularization or limb amputation. PAD is also associated with coronary artery disease (CAD) and is a strong predictor of poor cardiovascular outcomes [3]. Specifically, patients with PAD have a cardiovascular mortality rate of approximately 20% within 5 years of diagnosis [3,4].

Early identification of patients with PAD is critical for effective management. A combination of medical history taking, physical examination, and non-invasive tests such as the ankle brachial index (ABI) is the current gold standard for the diagnosis of PAD [2]. However, ABI measurements are operator-dependent and often falsely elevated in patients with comorbidities such as diabetes and chronic kidney disease [5]. Therefore, identification of better diagnostic and prognostic markers for PAD may improve risk-stratification and allow clinicians to select patients who require further diagnostic evaluation, close follow-up, and aggressive medical therapy. Furthermore, high risk patients may benefit from early surgical revascularization as a therapeutic target to improve blood flow to the lower extremities. Given that PAD diagnosis and treatment is often delayed, early identification and management of patients with PAD using biomarkers may improve their prognosis. In particular, aggressive risk factor medication, medical management, and referral to specialists have been demonstrated to improve limb salvage rates.

Recent literature has suggested that inflammation plays a crucial role in the initiation and progression of atherosclerotic disease [1,6]. In particular, interleukins (ILs) are a group of cytokines that have a wide variety of functions including pro-inflammatory, anti-inflammatory, and atherogenic effects [7]. These cell-signaling proteins are released in response to an inflammatory state for host defense [7]. Pro-inflammatory cytokines triggered by cholesterol crystals have been demonstrated to activate endothelial cells, thereby promoting the migration of monocytes, lymphocytes, and neutrophils, ultimately contributing to endothelial dysfunction in the early stages of atherosclerosis [7]. In addition, cytokines induce proliferation and migration of smooth muscle cells, which stimulates production and secretion of IL-1β by neutrophils, leading to a cascade of inflammation that contributes to atherosclerosis [7]. Moreover, in the later stages, pro-inflammatory cytokines contribute to thrombus formation by destabilizing atherosclerotic plaque through matrix degradation and apoptosis [7]. Most studies on ILs in atherosclerosis have been performed in isolation, and there exists no comprehensive review of the role of ILs in PAD. Consolidating our understanding of the role of inflammation in PAD could facilitate the development of novel diagnostics, management, and therapeutics for this disease in its early stages to improve patient outcomes. Given the significant role that inflammation plays in atherosclerotic development and progression, we hypothesized that there would be elevated levels of pro-inflammatory and pro-atherogenic ILs and downregulated levels of anti-inflammatory ILs in PAD. In this review, we summarized and critically evaluated the current literature investigating the role of ILs in PAD and their potential as diagnostic and prognostic biomarkers for PAD.

## 2. Methods

### 2.1. Design

A systematic review was conducted according to the Preferred Reporting Items for Systematic Reviews and Meta-Analyses (PRISMA) statement guidelines. The review was not registered.

### 2.2. Information Sources and Search Strategy

MEDLINE was searched from inception to 5 December 2022 for studies assessing the association between ILs and PAD. A combination of Medical Subject Heading (MeSH) terms, keywords, and synonyms for IL and PAD were used to maximize sensitivity. EndNote Version 20 was used to collate references [8]. We hand-searched the reference lists of included studies for additional relevant articles. The search strategy is detailed in Table A1.

### 2.3. Study Selection and Data Collection

Title and abstract screening, full-text review, and data collection were conducted by two independent reviewers, with a third author resolving discrepancies. Covidence was used to facilitate the systematic review [9]. We included all original studies assessing the association between ILs and PAD. Reviews, commentaries/editorials/letters, animal studies, papers analyzing non-PAD cardiovascular diseases (coronary artery disease, abdominal aortic aneurysm, and carotid artery stenosis), studies that did not directly measure IL levels, and articles without full text were excluded. A standardized form was used to collect data for included studies. Variables obtained were study authors, publication year, sample size, biomarker studied, protein expression levels, and key findings. Authors were contacted through email for relevant information not reported in the original publication.

### 2.4. Data Analysis

Due to the heterogeneity of the included studies, we did not conduct a meta-analysis. Therefore, a qualitative analysis summarizing key associations between studied ILs and PAD was performed.

## 3. Results

### 3.1. Study Screening and Selection

**Figure 1:**
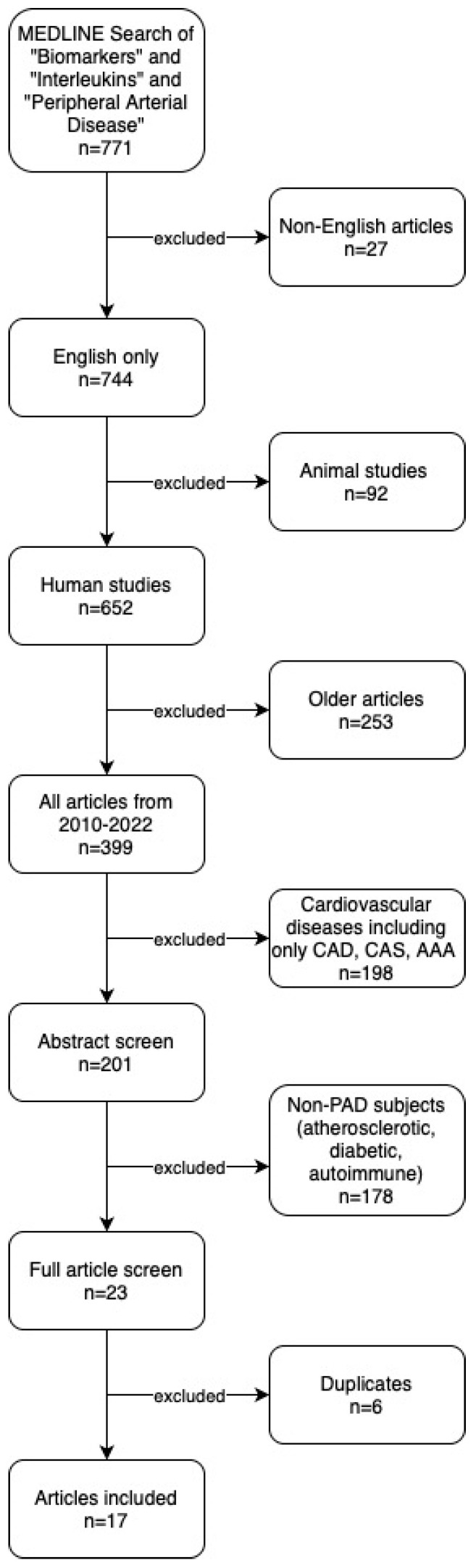
**PRISMA study flow diagram.** CAD: Coronary Artery Disease. CAS: Carotid Artery Stenosis. AAA: Abdominal Aortic Aneurysm.

We identified 771 articles through our search of MEDLINE and the following articles were excluded: non-English (*n* = 27), animal studies (*n* = 92), articles published before 2010 (*n* = 253), cardiovascular disease [CAD, CAS, AAA] (*n* = 198), non-PAD (*n* = 178), and duplicates (*n* = 6). Hand-search of reference lists identified no additional articles. In total, 17 studies were included in the final systematic review and qualitative analysis. Our search results are summarized in the PRISMA study flow diagram (Figure 1).

### 3.2. Study Characteristics

We included 17 studies published between 2010 and 2022. Studies measured plasma levels of the ILs in various PAD cohorts, from early stages of intermittent claudication to chronic limb threatening ischemia requiring intervention. In some studies, IL levels were measured both pre- and post-intervention, from 24 h up to 6 months post intervention. Most studies measured IL levels in addition to other pro-inflammatory markers such as tissue necrosis factor alpha (TNF-α) and ferritin to assess for any inflammatory state correlation. Pro-inflammatory cytokines IL-6 and IL-8 and anti-inflammatory cytokine IL-10 had the greatest number of studies undertaken compared to other ILs. A summary of our findings from the included studies can be found in Table 1.

### 3.3. Qualitative Analysis

**Figure 2:**
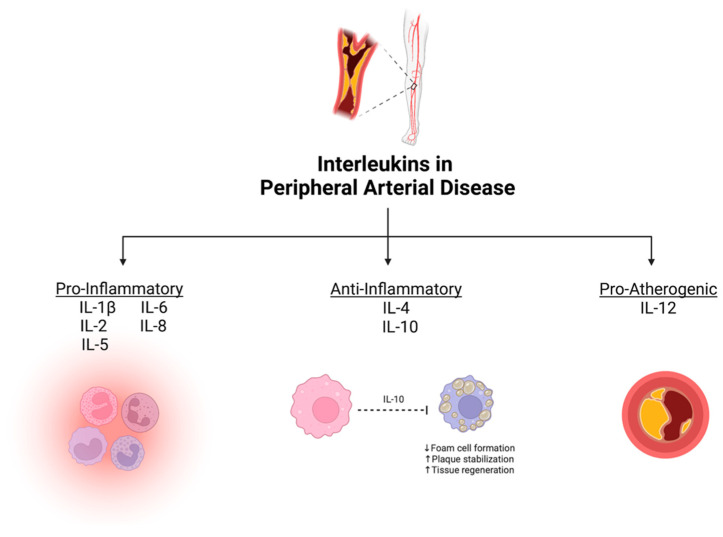
Summary of mechanistic associations between interleukins and peripheral artery disease. Created with BioRender.com.

From our systematic review, eight ILs were demonstrated to have potential as diagnostic/prognostic biomarkers for PAD. They were divided based on their primary physiological function into three groups: (1) pro-inflammatory ILs, defined as cytokines that accelerate atherosclerosis (IL-1β, IL-2, IL-5, IL-6, and IL-8); (2) anti-inflammatory ILs, defined as protective cytokines that help mitigate atherosclerosis (IL-4 and IL-10); and (3) pro-atherogenic ILs, defined as cytokines that enhance atherosclerotic plaque development (IL-12) [27]. A summary of the association between various ILs and PAD is depicted in Figure 2.

### 3.4. Key Findings: Pro-Inflammatory Cytokines

#### 3.4.1. Interleukin-6 (IL-6)

IL-6 has been heavily studied and linked to the inflammatory process throughout the many stages of atherosclerosis [25]. First, IL-6 stimulates the recruitment of leukocytes and their ability to transverse into the vessel wall by enhancing the expression of intercellular adhesion molecule 1 (ICAM-1) on the endothelium. It also induces the coagulation cascade by upregulating the surface expression of tissue factor on monocytes [25]. In advanced stages, IL-6 enhances the growth of atherosclerotic lesions by promoting the growth of smooth muscle cells through platelet-derived growth factor [25].

Studies have demonstrated that IL-6 plays a pro-atherogenic role throughout atherosclerosis by promoting thrombus formation and arterial occlusion [25]. IL-6 facilitates the release of acute phase reactant fibrinogen which promotes platelet aggregation and increases the expression of P-selectin which enhances platelet activation, promoting thrombus formation [25].

Furthermore, the initiation of platelet attachment to neutrophils and monocytes has also been linked to the activation of P-selectin by IL-6 enabling leukocyte-platelet binding [28,29].

In plasma, IL-6 was found to be elevated in asymptomatic PAD patients across various ethnic groups [16,17]. Several studies have investigated IL-6′s association with PAD prognosis [19]. One study conducted over three years compared high and low levels of IL-6, demonstrating that patients with elevated levels had a faster functional decline after measuring their walking performance [14]. However, a 2012 study revealed no significant change in IL-6 levels in intermittent claudication patients, over a one year period of supervised exercise training [15]. The Edinburgh Artery Study compared IL-6 to the leading inflammatory marker CRP and found IL-6 to be a superior earlier predictor of worsening ABI value when followed over 12 years [30]. Another group demonstrated that IL-6 was positively correlated with acute phase reactant ferritin, a protein known to be elevated in acute and chronic inflammation and associated with all-cause mortality. IL-6 and ferritin’s positive correlation suggests a potential mechanism for the increased risk of cardiovascular mortality in patients with PAD [24].

IL-6′s prognostic role has been extensively studied post-revascularization. Research has shown elevated levels of IL-6 in patients post peripheral, coronary, and cerebrovascular angioplasty with stent intervention. These post-revascularization findings suggesting high levels of platelet activation in atherosclerotic cardiovascular disease are in part due to IL-6′s heavy association with platelet P-selectin expression, GPIIb/IIIa activation, and leukocyte-platelet interaction [28]. Given the association between IL-6 and platelet activation, several studies have evaluated IL-6′s ability to predict in-stent restenosis in PAD patients who have undergone percutaneous transluminal angioplasty (PTA) with stent implantation. In 2015, one study found elevated IL-6 levels immediately post-procedure, with steady increase for up to 24 h [18]. Similarly, another study found elevated IL-6 levels 36 h post-endovascular intervention for patients with PAD, again depicting an inflammatory status post-intervention [20]. In a comparative study between IL-6 and CRP, plasma levels were measured at baseline and 24-h post-femoropopliteal artery angioplasty with stenting to predict restenosis at 6 months. IL-6 showed superior predictive capability for in-stent restenosis at 6 months compared to CRP [23]. Lastly, one study measured serum blood levels of IL-6 along with ultrasound brachial artery flow-mediated dilatation (FMD) pre- and post-femoropopliteal bypass grafting to identify changes in endothelial function [21]. After revascularization, the endothelium function improved as the FMD level increased and correlated with the decrease in IL-6 levels [13]. These findings suggest an association between IL-6 and inflammation contributing to graft/stent occlusion.

#### 3.4.2. Interlukin-8 (IL-8)

IL-8 is a pro-inflammatory IL that is typically released by monocytes and macrophages. IL-8 is involved in the recruitment of monocytes and neutrophils in the acute inflammatory response. High levels of IL-8 have been found in atherosclerotic arterial walls. IL-8 has also been identified as an independent predictor of cardiovascular events in patients with coronary artery disease (CAD) [31]. Monocyte-derived macrophages have been implicated in the development of atherosclerotic plaque formation and IL-8 plays a crucial role in regulating the endothelial interaction of neutrophils. During exercise-induced ischemia, cytokines and cell adhesion molecules (CAM) allow for the migration of adhered neutrophils through the endothelium. IL-8 has been shown to increase the surface expression of CAM, thereby contributing to atherosclerosis. However, a 2014 study revealed high levels of IL-8 and TNF-α in PAD group, without worse endothelial measures [10].

A 1999 study investigated IL-8 levels in patients with and without PAD. Serum IL-8 levels were measured before a standard acute treadmill-exercise test and in 5-min intervals thereafter (1, 5, and 10). PAD patients showed significantly higher levels of IL-8 both before and after the exercise test. IL-8 levels post 5 min exercise were significantly different between study and control groups, with a 53% change from pre-exercise in the control group and a 5% change in the PAD population. Authors attributed elevated levels of IL-8 and subsequent failure of levels to fall by the same extent after exercise in the PAD group to increased neutrophil activation, reduced blood flow, and increased cytokine production during ischemia–reperfusion [32].

A 2019 study conducted by Gremmels et al. reported four cytokines, IL-8, IL-6, growth-related oncogene-alpha (GRO-α), and Interferon-gamma Inducible Protein-10 (IP-10), as predictors of major adverse limb events in patients with chronic limb threatening ischemia. Of these four, IL-8, IL-6, and GRO-α, were found to have a high degree of correlation with amputation. The authors suggest that this finding was likely due to the co-regulation of these three proteins in the acute inflammatory response [22].

Similarly, a 2015 study by Arajao et al. evaluated the relationship between a panel of biomarkers and the development of in-stent restenosis prior to femoral PTA with stent implantation, as well as 24 h and 6 months after. The authors reported a statistically significant reduction in IL-8 at 24 h after versus pretreatment, 6 months vs. pretreatment, and 6 months vs. 24 h. A trend was reported of increased levels of IL-6, TNF-α, transforming growth factor beta (TGF-β), and IL-12 at 24 h after PTA and stenting compared with pretreatment, irrespective of restenosis [18]. These findings suggest that stent implantation triggers a pro-inflammatory response, thereby contributing to the risk of restenosis/occlusion.

#### 3.4.3. Interlukin-1β (IL-1β)

IL-1β is a cytokine that belongs to the IL-1 family and has two isoforms. IL-1α is typically found on the cell surface and acts at short distances via direct contact, while IL-1β acts at a distance. IL-1β signaling is transduced by the IL-1 Receptor I, while IL-1 Receptor II (IL-1RII) provides negative regulation. IL-1α or IL-1β may act on themselves or each other, to create a positive feedback loop, or increase the expression of IL-1ra, resulting in negative feedback inhibition. This family of cytokines activate mononuclear phagocytes and increase the expression of leukocyte adhesion molecules and thrombogenic mediators. IL-1β has gained attention as a therapeutic target for atherosclerosis, particularly from the selective neutralization of IL-1β by canakinumab, a monoclonal antibody [33]. A 2019 study reported that there was no significant change in superficial femoral artery plaque burden in PAD patients treated with canakinumab. The authors concluded that the treatment may improve both maximum and pain-free walking distance in patients with symptomatic PAD, thereby suggesting functional improvement in PAD symptoms from IL-1β inhibition [34].

#### 3.4.4. Interlukin-2 (IL-2)

IL-2 is a well-studied IL and one of the first to be discovered. It is produced by activated CD4+ T-helper cells to support CD8+ T cells with respect to the generation of memory T cells. IL-2 also has some anti-inflammatory capacity by inhibiting IL-17 production in T-helper 17 cells [35]. In a 2009 study profiling circulating cytokine levels in PAD patients, no significant difference in IL-2 levels was observed between stable and intermittent claudication populations. This study attributed a limited role of IL-2 and IL-1β in the pathophysiology of PAD due to their low levels in both study groups [36].

Another 2010 study explored the relationship between iron stores and interleukin levels over a 6-year period among 100 patients with PAD. The trial’s aim was to test if a reduction in the iron stores of PAD patients could improve clinical outcomes, as excess iron levels promote oxidative-stress-induced inflammation in PAD [11]. To test this hypothesis, the study analyzed the correlation of inflammatory biomarker levels and ferritin, an acute phase reactant, through phlebotomy-induced iron store reduction. No correlation was found between IL-2 levels and ferritin levels, measured at entry and at 6-month intervals for 6 years. Therefore, the potential role of IL-2 as a diagnostic/prognostic biomarker for PAD is limited.

### 3.5. Key Findings: Anti-Inflammatory Cytokines

Anti-inflammatory cytokines counteract pro-inflammatory cytokines to maintain immune homeostasis and prevent a state of chronic inflammation. They play a crucial role in resolving inflammation, prevent excessive tissue damage, and promote tissue repair in atherosclerotic lesions. They can suppress the production of pro-inflammatory cytokines, inhibit chemotaxis of pro-inflammatory macrophages and T-cells to inflammatory sites, and prevent the formation of foam cells by inhibiting lipid accumulation inside macrophages [27]. For these few roles mentioned, anti-inflammatory cytokines are known for their protective effects on atherosclerosis. The following ILs were demonstrated to be suppressed in patients with PAD.

#### 3.5.1. Interlukin-10 (IL-10)

IL-10 is believed to have a protective role in atherosclerosis, particularly in the formation and stability of atherosclerotic plaque [37]. This anti-inflammatory cytokine is a key player in the human immune response. Upon interaction with its cellular receptor, this homodimer of two tightly packed 160-amino-acid proteins inhibits cytokine production in neutrophils, natural killer cells, monocytes, macrophages, and T helper-type 1 (Th1) lymphocyte responses [38]. Inhibition of Th1 responses prevents the release of IL-2 and interferon gamma (IFN-γ) from Th1 lymphocytes. It is due to this function that IL-10 was labeled as a cytokine synthesis inhibition factor. Cytokines produced by monocytes and macrophages include TNF-α, IL-1, IL-6, IL-8, IL-12, granulocyte colony-stimulating factor, Macrophage inflammatory protein-1-alpha (MIP-1α), and Macrophage inflammatory protein-2-alpha (MIP-2α) [38].

In a 2010 study by Marghani et al., IL-10 and mitogen-activated protein kinases-p38 (MAPK p38) levels were compared in patients with intermittent claudication, with the perspective that PAD is a predictor for cardiovascular disease morbidity and mortality [12]. This paper included a study group of patients with stable angina, a group that has previously shown itself to have significantly higher serum levels of IL-10 compared to those with unstable angina [39]. These two molecules were analyzed together, with the hypothesis that intracellular signaling molecule MAPK p38 is down regulated by IL-10, controlling inflammatory activity in stable angina. Among their study groups, serum levels of IL-10 were highest in the stable angina group, compared to intermittent claudication and control groups. Additionally, there were significantly elevated levels of MAPK p38 in PAD patients [39].

Similarly, another protein studied together with IL-10 was alpha-Klotho (α-Klotho), which has anti-inflammatory properties and is associated with protective mechanisms in cardiovascular diseases. Where IL-10 acts on monocytes and macrophages, α-Klotho is expressed in these cells. There is an assumption that α-Klotho levels are controlled in a negative-feedback fashion, as pro-inflammatory processes can reduce its expression. The study consisted of 76 patients who participated in the case group and had an elective open vascular surgery procedure due to clinical atherosclerotic vascular disease, where 45 of these patients had PAD and intermittent claudication. Serum level of IL-10 was found to be reduced in the case group, and there was a direct association between circulating levels of IL-10 and α-Klotho gene expression in peripheral blood circulating cells (PBCCs). This is of note, as the adhesion of PBCCs to the vascular wall is an initial step in the atherogenic process. A parameter for assessing global inflammatory status was calculated by the group as the ratio of TNF-α and IL-10, which showed an inverse relation to α-Klotho gene expression [26]. This work highlights the protective effect of IL-10 in atherosclerosis.

#### 3.5.2. Interlukin-4 (IL-4)

IL-4 is an anti-inflammatory cytokine produced in T-helper-type 2 (Th2) cells and is assumed to have a protective role in atherosclerosis for this reason [12]. IL-4 activity is most widely recorded in aortic aneurysms. This is supported by IL-4′s role in causing increased expression of P-selectin, vascular cell adhesion molecule-1 (VCAM-1), and Matrix Metalloproteinase (MMP) 1 and 12, known to be implicated in the formation of aortic aneurysms. Previous studies have reported some evidence of IL-4 in acute coronary syndrome, however in the case of PAD, cross-sectional studies in a smaller scale only reported non-significant variations in IL-4 levels in association with PAD [40]. Therefore, the role of IL-4 in PAD may be limited.

### 3.6. Key Findings: Pro-Atherogenic Cytokines

#### Interleukin-12 (IL-12)

Interleukin-12, which is secreted by monocytes, is known to be a T-cell growth factor as it induces the differentiation of naïve T-cells into Th1 cells [18]. Th1 cells secrete IFN-γ which stimulates macrophages and contributes to advancing atherosclerosis [41]. Thus, IL-12 has been implicated as a pro-atherogenic cytokine that plays an integral role in the generation and progression of atherosclerotic plaque development [41]. It is hypothesized that the balance between IL-12 and IL-10 contributes to the extent of tissue damage mediated by the immune system in atherosclerosis. This is supported by the findings that IL-10 can downregulate IL-12. If this regulatory pathway is not carefully balanced, it can have an impact on the inflammatory cascade involved in atherosclerosis [42]. In a 2015 study by Araujo et al., plasma markers were examined for in-stent restenosis in PAD patients 24 h post-intervention and at 6 months follow up. IL-12 levels were found to be elevated 24 h after PTA and stenting, showing a new possible relation with inflammation in addition to its atherogenic effects [18].

## 4. Discussion

### 4.1. Summary of Findings

This systematic review and qualitative analysis of 17 studies published over a 12-year period provides a comprehensive synthesis and rigorous evaluation of the literature assessing the association between ILs and PAD. We found five pro-inflammatory ILs (IL-1β, IL-2, IL-5, IL-6, and IL-8) and one pro-atherogenic IL (IL-12) that were positively correlated with PAD diagnosis and prognosis. In contrast, two anti-inflammatory ILs (IL-4 and IL-10) were downregulated in PAD.

Inflammation plays a major role in the initiation and chronic nature of systemic atherosclerosis which contributes to PAD. ILs are cytokines with diverse functions including both promoting and reducing inflammation, in addition to the initiation and progression of atherosclerosis. Some well-known pro-inflammatory cytokines such as IL-1, TNF-α, and IL-6 exert effects such as promoting endothelial dysfunction, monocyte migration, smooth muscle cell proliferation, thrombus formation, and plaque destabilization. Conversely, anti-inflammatory cytokines such as IL-10 offer protective effects by means of suppressing inflammatory pathways and regulating the immune response.

In our systematic review, we analyzed the association of ILs and their ability to diagnose and predict outcomes in patients with PAD. We found that serum levels of pro-inflammatory and pro-atherogenic ILs were elevated in PAD, while anti-inflammatory ILs were decreased. Results demonstrate that few ILs have been studied, uncovering a gap in our knowledge that can be used to personalize medicine in PAD.

### 4.2. Comparison to Existing Literature

This is the first comprehensive review of the association between ILs and PAD. Extensive research has been conducted on pro-inflammatory cytokine IL-6 with regards to its role in inflammation and atherosclerosis. IL-6 mediates leukocyte infiltration, activation of the clotting cascade, stimulating the growth of smooth muscle cells, and release of acute phase reactants. Studies have found elevated IL-6 levels in asymptomatic undiagnosed PAD patients and diabetic patients with limb arterial stenosis. Furthermore, IL-6 has been shown to be a promising prognostic marker for predicting functional decline, worsening ABI status, and future cardiovascular events. Gremmels et al. created a more accurate prediction model by combining IL-6 with another prognostic inflammatory marker, IP-10, for patients with chronic limb-threatening ischemia to predict major amputation and death. In advanced stages of PAD, IL-6 has been associated with complications such as in-stent restenosis and alterations in endothelial function. Their model significantly improved prognostic accuracy by a striking twelvefold, demonstrating the potential future direction for IL-6 as a marker for PAD [22]. Therefore, combining IL-6 with other inflammatory markers can assist in creating a tool to assist clinicians in the identification of high-risk patients and tailor management after surgical interventions to improve patient outcomes [22].

Anti-inflammatory cytokine IL-4 has been well studied in acute coronary syndrome and AAA; however, it is not as thoroughly explored in PAD. In the small number of studies that have investigated IL-4 in patients with PAD, variation of IL-4 levels among groups was reported to be insignificant. This was consistent with our review findings where the same study reported significant variation in IL-10. This study, investigating MAPK p38 levels, also reported a significantly high level of IL-5, another IL not well studied in PAD. As with IL-2, past studies showed no significance in its variation, likely attributed to its limited role in the development of PAD. This trend was also observed in our study.

### 4.3. Implications

This systematic review provides valuable insight into studies exploring the potential for ILs to be used as diagnostic and prognostic biomarkers for PAD. Specifically, IL-8 was strongly associated with in-stent restenosis post-PTA and stenting in PAD patients. Two studies from 2015 and 2018 tracked the cytokine’s expression at various points after stenting and additionally tracked restenosis and its correlation to the cytokine expression. In both studies, a significant reduction in IL-8 levels was observed post-intervention [18]. Furthermore, IL-8 and IL-6 had a high correlation with future amputation, suggesting the protein’s ability to predict adverse PAD-related events [22].

Studies in our search presented the trend of conducting biomarker evaluation in panels. This is particularly useful in studying levels of anti-inflammatory interleukins such as IL-10 in order to contrast the levels of pro-inflammatory cytokines such as IL-12. Atherosclerosis is believed to stem from a fine balance between pro- and anti-inflammatory cytokines. Disruption of this balance, where inflammation fails to resolve, leads into a chronic inflammatory state resulting in tissue damage and atherosclerotic plaque formation, ultimately progressing to PAD. For these reasons, cytokines such as IL-10 are necessary for downregulating IL-12 to restore and improve the immune system’s state of homeostasis [42]. Therefore, strengthening our understanding of the mechanistic relationships between inflammatory proteins and PAD may allow us to identify novel diagnostic/prognostic biomarkers for this debilitating disease.

### 4.4. Limitations

This study has several limitations. First, a meta-analysis was not conducted due to heterogeneity between outcomes measured in the included studies. Second, there may be publication bias, with positive studies being more likely to be published. Third, our goal was to review contemporary studies; papers published prior to 2010 were excluded.

## 5. Conclusions

Our systematic review and qualitative analysis of 17 studies highlights the diagnostic and prognostic potential of ILs in PAD. In particular, IL-6 and IL-8 are pro-inflammatory cytokines associated with PAD development and progression. Furthermore, patients with PAD have been found to have high plasma levels of IL-6, making it a target in the prevention and treatment of PAD, especially in the asymptomatic population and in predicting future cardiovascular events. Ongoing work to identify and validate diagnostic and prognostic markers for PAD has the potential to assist clinicians in identifying high-risk patients earlier for diagnosis and treatment which could reduce the risk of adverse cardiovascular and limb events.

## Figures and Tables

**Table 1:** Summary of included articles assessing the association between interleukins and peripheral artery disease.

Title	Study	Population	Biomarker	Expression in Patients with PAD	Findings
**Pro-Inflammatory**					
Impaired vascular endothelial growth factor A and inflammation in patients with peripheral artery disease	Gardner et al., 2014 [10]	136 PAD	IL-1β	No change	There was no statistically significant difference in the serum levels between controls and PAD group (*p* = 0.618). Median serum levels of IL-1β in PAD patients 15.8 pg/mL vs. controls 16.0 pg/mL).
Critical analysis and limitations of resting ankle-brachial index in the diagnosis of symptomatic peripheral arterial disease patients and the role of diabetes mellitus and chronic kidney disease	Guimaraes et al., 2018 [5]	27 PAD	IL-1β	Decreased	IL-1β (pg/mL) showed reduced levels, when comparing pre-treatment levels vs. 24 h after (*p* < 0.01), pre-treatment levels vs. 6 months after (*p* < 0.01), and 24 h after vs. 6 months after (*p* < 0.01).
Ferritin levels, inflammatory biomarkers, and mortality in peripheral arterial disease: a substudy of the Iron (Fe) and Atherosclerosis Study (FeAST) Trial	Depalma et al., 2010 [11]	100 PAD	IL-2	Not reported	No correlation was found between IL-2 levels and ferritin levels, measured at entry and at 6-month intervals for 6 years (*p* = 0.46).
High MAPK p38 activity and low level of IL-10 in intermittent claudication as opposed to stable angina	Marghani et al., 2010 [12]	5 IC	IL-5	Elevated	Levels in intermittent claudication group was upregulated (*p* = 0.0047) compared to controls.
Ferritin levels, inflammatory biomarkers, and mortality in peripheral arterial disease: a substudy of the Iron (Fe) and Atherosclerosis Study (FeAST) Trial	Depalma et al., 2010 [11]	51 PAD	IL-6	Elevated	Results showed patients who died had elevated ferritin and IL-6 levels.
High MAPK p38 activity and low level of IL-10 in intermittent claudication as opposed to stable angina	Marghani et al., 2010 [12]	5 IC	IL-6	Elevated	Levels in intermittent claudication group were upregulated (*p* = 0.0026) compared to controls.
Effects of lower extremity revascularization on the endothelial functions measured with noninvasive brachial artery flow-mediated dilatation	Unal et al., 2011 [13]	54 PAD	IL-6	Decreased	Serum levels of IL-6, nitric oxide, hs-CRP, and TNF-α levels were measured pre-and post-op. IL-6 levels measured pre- 49.1 ± 8.4 mg/mL and post-op 43.9 ± 8.8 nmol/L were found to be significantly decreased 4 weeks following femoropopliteal bypass. The study concluded endothelial function (measured by using non-invasive brachial artery flow mediated dilatation (FMD)) dramatically improved following revascularization.
Relation of interleukin-6 and vascular cellular adhesion molecule-1 levels to functional decline in patients with lower extremity peripheral arterial disease	McDermott et al., 2011 [14]	255 PAD	IL-6	Elevated	Results showed consistent high levels of IL-6 are associated with faster functional decline compared to low or fluctuating levels. Soluble vascular adhesion molecule (sVCAM-1) showed no significance at any level.
Impact of exercise training on inflammation and platelet activation in patients with intermittent claudication	Schlager et al., 2012 [15]	54 IC	IL-6	No change	IL-6 was one of the markers assessed at baseline, 3-, 6-, and 12-months’ time. No significant changes in markers were found with the addition of supervised exercise training (SET) to best medical therapy.
Patients with unrecognized peripheral arterial disease (PAD) assessed by ankle-brachial index (ABI) present a defined profile of proinflammatory markers compared to healthy subjects	Signorelli et al., 2012 [16]	71 PAD	IL-6	Elevated	IL-6 was found elevated (*p* < 0.0001) and transforming growth factor-b1 (TGF-b1; anti-inflammatory cytokine) decreased (*p* < 0.0001) in PAD patients compared to controls.
Associations of candidate biomarkers of vascular disease with the ankle-brachial index and peripheral arterial disease	Ye et al., 2013 [17]	2443 PAD	IL-6	Elevated	Exactly 47 biomarkers proved to be different in the different ethnic groups. IL-6 was elevated and associated with the presence of PAD in the African American group, and none of the 47 markers were associated with PAD in the non-Hispanic white (NHW) group.
Impaired vascular endothelial growth factor A and inflammation in patients with peripheral artery disease	Gardner et al., 2014 [10]	136 PAD	IL-6	Elevated	IL-1β, IL-6, IL-8, tissue necrosis factor alpha (TNF-α), monocyte chemotactic protein-1 (MCP-1), hepatocyte growth factor (HGF), nerve growth factor (NGF), serum amyloid A (SAA), vascular endothelial growth factor-A (VEGF-A), and adiponectin levels were assessed.There was no statistically significant difference in the serum levels between controls and the PAD group (*p* = 0.246). Median serum levels of IL-6 in the PAD group were 23.0 pg/mL vs. controls 21.0 pg/mL.
Interleukins and inflammatory markers in in-stent restenosis after femoral percutaneous transluminal angioplasty	Araujo et al., 2015 [18]	26 PAD	IL-6	Elevated	IL-6 levels increased twofold 24 h post-percutaneous transluminal angioplasty (PTA) and decreased 6 months later (*p* < 0.05). IL-6 was not found to be a predictor for in-stent restenosis 6-months post PTA with stenting.
Plasma Levels of Inflammatory Biomarkers in Peripheral Arterial Disease: Results of a Cohort Study	Signorelli et al., 2016 [19]	80 PAD	IL-6	Elevated	Serum markers assessed were interleukin 6 (IL-6), tumor necrosis factor a (TNF-a), L-selectin (LS), neopterin (N), P-selectin (PS), E-selectin (ES), vascular cell adhesion molecule 1 (VCAM-1), intercellular adhesion molecule 1 (ICAM-1), and matrix metalloproteinase 2 (MMP-2) and 9 (MMP-9). Mean serum concentration of IL-6 in patients with PAD was 11.8 + 1.2 ng/dL. All 10 inflammatory markers assessed were found to be significantly elevated in patients with PAD [IL-6 (*p* < 0.001)].
Circulating malondialdehyde-modified low-density lipoprotein (MDA-LDL) as a novel predictor of clinical outcome after endovascular therapy in patients with peripheral artery disease (PAD)	Takamura et al., 2017 [20]	35 PAD	IL-6	Elevated	The following markers were investigated IL-6, hs-CRP, D-dimer, and malondial- dehyde-modified low-density lipoprotein [MDA-LDL]. Mean serum levels of IL-6, hs-CRP, and D-dimer increased and were significantly elevated 36 h following intervention.
The Role of Interleukins and Inflammatory Markers in the Early Restenosis of Covered Stents in the Femoropopliteal Arterial Segment	Guimaraes et al., 2018 [21]	27 PAD	IL-6	Decreased	The following cytokines IL-1β, IL-6, IL-8, and IL-10 were examined for in-stent restenosis 6 months post intervention. Serum levels of IL-6 decreased 24 h and 6 months post angioplasty with stenting (*p* < 0.01).
A Pro-Inflammatory Biomarker-Profile Predicts Amputation-Free Survival in Patients with Severe Limb Ischemia	Gremmels et al., 2019 [22]	118 PAD and 108 CLTI	IL-6	Elevated	IL-6, IL-8, GROα, and IP-10 were found to be predictive of major events in CLTI patients. IL-6 showed itself to be a promising predictor of amputation rather than mortality with HR of 1.58 (95% CI: 1.24–2.0, *p* = 0.0002).IL-6 and IP-10 together demonstrated a twelvefold increase in predictive ability for negative outcomes. The authors created a prediction model with 78% more accuracy to risk-stratify patients with CLTI.
Six-month results of stenting of the femoropopliteal artery and predictive value of interleukin-6: Comparison with high-sensitivity C-reactive protein	Guo et al., 2020 [23]	68 PAD	IL-6	Elevated	Pre- and post-intervention serum levels of IL-6 are compared to hs-CRP to determine predictive value of in-stent restenosis. Results showed IL-6 levels were elevated at baseline and 24 h post-intervention and demonstrated greater prognostic value for re-stenosis at 6 months compared to hs-CRP.
Optimal serum ferritin level range: iron status measure and inflammatory biomarker	DePalma RG et al., 2021 [24]	100 PAD	IL-6	Elevated	The results suggest that ferritin serum levels coincide with IL-6 serum levels. Specifically, higher serum ferritin and IL-6 levels are associated with increased mortality in patients with PAD.
Discovery of four plasmatic biomarkers potentially predicting cardiovascular outcome in peripheral artery disease	Kremers et al., 2022 [25]	120 PAD	IL-6	Elevated	Four proteins IL-6, PAR1, TNFRSF11A, and Gal-9 were assessed. Interleukin-6 showed high predictive value with a hazard ratio of 2.02 [1.35–3.02] for cardiovascular events and mortality in patients with PAD.
Impaired vascular endothelial growth factor A and inflammation in patients with peripheral artery disease	Gardner et al., 2014 [10]	130 PAD	IL-8	Elevated	IL-8 levels were found to be significantly higher (*p* = 0.006) in the study group compared to controls. Median serum levels of IL-8 in PAD patients equaled 94.0 pg/mL vs. controls 77.5 pg/mL.
C-reactive protein, interleukin-6, and soluble adhesion molecules as predictors of progressive peripheral atherosclerosis in the general population: Edinburgh Artery Study	Araújo et al., 2015 [18]	26 PAD	IL-8	Decreased	IL-8 levels showed a statistically significant reduction 24 h after vs. pre-treatment (*p* < 0.05), 6 months vs. pre-treatment (*p* < 0.05), and 6 months vs. 24 h (*p* < 0.01).
The Role of Interleukins and Inflammatory Markers in the Early Restenosis of Covered Stents in the Femoropopliteal Arterial Segment.	Guimaraes et al., 2018 [21]	27 PAD	IL-8	Decreased	IL-8 showed reduced levels when comparing pre-treatment levels vs. 6 months after (*p* < 0.01) and 24 h after vs. 6 months after (*p* < 0.01).
Interleukin 8 and cardiovascular disease	Gremmels et al., 2019 [22]	118 PAD and 108 CLTI	IL-8	Decreased	IL-8 showed itself to be predictive of amputation rather than mortality with HR 1.93 (95% CI: 1.3–2.9, *p* = 0.002) for IL-8.
**Anti-Inflammatory**					
Protective Role of Interleukin-10 in Atherosclerosis	Marghani et al., 2010 [12]	5 IC	IL-4	Not reported	Levels in intermittent claudication group not reported. IL-4 level in the stable angina group was significantly elevated (*p* = 0.008) compared with the healthy control group.
The Role of Interleukins and Inflammatory Markers in the Early Restenosis of Covered Stents in the Femoropopliteal Arterial Segment.	Guimaraes et al., 2018 [21]	27 PAD	IL-10	Elevated	IL-10 levels steadily increased 24 h and 6 months post-intervention (*p* < 0.01).
Protective Role of Interleukin-10 in Atherosclerosis	Marghani et al., 2010 [12]	5 IC	IL-10	Decreased	IL-10 level in the stable angina group was significantly elevated (*p* = 0.0116), compared with both the intermittent claudication group (*p* = 0.0317) and the healthy control group.
Ferritin levels, inflammatory biomarkers, and mortality in peripheral arterial disease: a substudy of the Iron (Fe) and Atherosclerosis Study (FeAST) Trial	Depalma et al., 2010 [11]	100 PAD	IL-10	Not reported	No correlation was found between IL-10 levels and ferritin levels, measured at entry and at 6-month intervals for 6 years (*p* = 0.48).
Plasma Levels of Inflammatory Biomarkers in Peripheral Arterial Disease: Results of a Cohort Study	Martín-Núñez et al., 2022 [26]	45 IC and PAD	IL-10	Decreased	IL-10 concentration in serum was reduced compared to control group. [3.93 (0.61 to 10.13) versus 10.38 (7.52 to 30.20), *p* < 0.001].Ratio of TNF-α and IL-10 was calculated to assess global inflammatory status. This parameter was significantly higher in the test group compared to the control group [0.28 (0.10 to 1.62) vs. 0.11 (0.02 to 0.27), *p* < 0.01].
**Pro-Atherogenic**					
C-reactive protein, interleukin-6, and soluble adhesion molecules as predictors of progressive peripheral atherosclerosis in the general population: Edinburgh Artery Study	Araújo et al., 2015 [18]	26 PAD	IL-12	Elevated	IL-12 levels were found elevated 24 h post-PTA (39.17 ± 135.6 (pg/mL)) and stenting when compared to pre-treatment (15.33 ± 22.6 (pg/mL)). Plasma concentration at 6-months post-treatment equaled 30.39 ± 101.0 (pg/mL). IL-12 was not found to be a predictor for in-stent restenosis 6-months post PTA with stenting.

IC: Intermittent claudication. PAD: Peripheral arterial disease. CLTI: Chronic limb threatening ischemia. PTA: Percutaneous Transluminal Angioplasty.

## Data Availability

The original contributions presented in this study are included in the article and further inquiries can be directed to the corresponding author.

## Appendix A

**Table A1:** Search strategy.

**Ovid MEDLINE(R) <1946 to December Week 2 2022>**		
1	exp Biomarkers/	865,584
2	exp Interleukins/	266,567
3	exp Arterial Occlusive Diseases/or peripheral arterial disease.mp. or exp Peripheral Arterial Disease/or exp Intermittent Claudication/or exp Peripheral Vascular Diseases/	306,540
4	1 and 2 and 3	771
5	limit 4 to English language	744
6	limit 5 to humans	652
7	limit 6 to yr = “2010–2022”	399
8	exp Coronary Artery Disease/	73,357
9	exp Aortic Aneurysm, Abdominal/	21,831
10	exp Carotid Stenosis/	17,657
11	(1 and 2 and 3 and 4 and 5 and 6 and 7) not 8 not 9 not 10	201

## References

[1] Bartoli-Leonard F., Zimmer J., Sonawane A.R., Perez K., Turner M.E., Kuraoka S., Pham T., Li F., Aikawa M., Singh S. (2023). NLRP3 Inflammasome Activation in Peripheral Arterial Disease. J. Am. Heart Assoc..

[2] Abramson B.L., Al-Omran M., Anand S.S., Albalawi Z., Coutinho T., De Mestral C., Dubois L., Gill H.L., Greco E., Guzman R. (2022). Canadian Cardiovascular Society 2022 Guidelines for Peripheral Arterial Disease. Can. J. Cardiol..

[3] Doobay A.V., Anand S.S. (2005). Sensitivity and Specificity of the Ankle–Brachial Index to Predict Future Cardiovascular Outcomes: A Systematic Review. Arterioscler. Thromb. Vasc. Biol..

[4] Tran A.T., Spertus J.A., Mena-Hurtado C.I., Jones P.G., Aronow H.D., Safley D.M., Malik A.O., Peri-Okonny P.A., Shishehbor M.H., Labrosciano C. (2022). Association of Disease-Specific Health Status with Long-Term Survival in Peripheral Artery Disease. J. Am. Heart Assoc..

[5] AbuRahma A.F., Adams E., AbuRahma J., Mata L.A., Dean L.S., Caron C., Sloan J. (2020). Critical Analysis and Limitations of Resting Ankle-Brachial Index in the Diagnosis of Symptomatic Peripheral Arterial Disease Patients and the Role of Diabetes Mellitus and Chronic Kidney Disease. J. Vasc. Surg..

[6] Libby P., Ridker P.M., Maseri A. (2002). Inflammation and Atherosclerosis. Circulation.

[7] Warnatsch A., Ioannou M., Wang Q., Papayannopoulos V. (2015). Neutrophil Extracellular Traps License Macrophages for Cytokine Production in Atherosclerosis. Science.

[8] (2021). EndNote Version 20.1 [Computer Software]. http://endnote.com.

[9] (2023). Covidence Systematic Review Software, [Computer Software]. https://www.covidence.org.

[10] Gardner A.W., Parker D.E., Montgomery P.S., Sosnowska D., Casanegra A.I., Esponda O.L., Ungvari Z., Csiszar A., Sonntag W.E. (2014). Impaired Vascular Endothelial Growth Factor A and Inflammation in Patients with Peripheral Artery Disease. Angiology.

[11] DePalma R.G., Hayes V.W., Chow B.K., Shamayeva G., May P.E., Zacharski L.R. (2010). Ferritin Levels, Inflammatory Biomarkers, and Mortality in Peripheral Arterial Disease: A Substudy of the Iron (Fe) and Atherosclerosis Study (FeAST) Trial. J. Vasc. Surg..

[12] Lee Y.-W., Kim P.H., Lee W.-H., Hirani A.A. (2010). Interleukin-4, Oxidative Stress, Vascular Inflammation and Atherosclerosis. Biomol. Ther..

[13] Unal O., Karatepe O., Ugurlucan M., Koc B., Filizcan U., Aksoy M. (2011). Effects of Lower Extremity Revascularization on the Endothelial Functions Measured with Noninvasive Brachial Artery Flow-Mediated Dilatation. Ann. Vasc. Surg..

[14] McDermott M.M., Liu K., Ferrucci L., Tian L., Guralnik J.M., Tao H., Ridker P.M., Criqui M.H. (2011). Relation of Interleukin-6 and Vascular Cellular Adhesion Molecule-1 Levels to Functional Decline in Patients with Lower Extremity Peripheral Arterial Disease. Am. J. Cardiol..

[15] Schlager O., Hammer A., Giurgea A., Schuhfried O., Fialka-Moser V., Gschwandtner M., Koppensteiner R., Steiner S. (2012). Impact of Exercise Trainting on Inflammation and Platelet Activation in Patients with Intermittent Claudication. Swiss. Med. Wkly..

[16] Signorelli S.S., Anzaldi M., Fiore V., Simili M., Puccia G., Libra M., Malaponte G., Neri S. (2012). Patients with Unrecognized Peripheral Arterial Disease (PAD) Assessed by Ankle-Brachial Index (ABI) Present a Defined Profile of Proinflammatory Markers Compared to Healthy Subjects. Cytokine.

[17] Ye Z., Ali Z., Klee G.G., Mosley T.H., Kullo I.J. (2013). Associations of Candidate Biomarkers of Vascular Disease with the Ankle-Brachial Index and Peripheral Arterial Disease. Am. J. Hypertens..

[18] Araújo P.V., Ribeiro M.S., Dalio M.B., Rocha L.A., Viaro F., Joviliano R.D., Piccinato C.E., Évora P.R.B., Joviliano E.E. (2015). Interleukins and Inflammatory Markers in In-Stent Restenosis after Femoral Percutaneous Transluminal Angioplasty. Ann. Vasc. Surg..

[19] Signorelli S.S., Anzaldi M., Libra M., Navolanic P.M., Malaponte G., Mangano K., Quattrocchi C., Di Marco R., Fiore V., Neri S. (2016). Plasma Levels of Inflammatory Biomarkers in Peripheral Arterial Disease: Results of a Cohort Study. Angiology.

[20] Takamura T., Tsuchiya T., Oda M., Watanabe M., Saito R., Sato-Ishida R., Akao H., Kawai Y., Kitayama M., Kajinami K. (2017). Circulating Malondialdehyde-Modified Low-Density Lipoprotein (MDA-LDL) as a Novel Predictor of Clinical Outcome after Endovascular Therapy in Patients with Peripheral Artery Disease (PAD). Atherosclerosis.

[21] Guimaraes T.S., Da Rocha L.A., Becari C., Piccinato C.E., Joviliano R.D., Ribeiro M.S., Joviliano E.E. (2018). The Role of Interleukins and Inflammatory Markers in the Early Restenosis of Covered Stents in the Femoropopliteal Arterial Segment. Ann. Vasc. Surg..

[22] Gremmels H., Teraa M., De Jager S.C.A., Pasterkamp G., De Borst G.J., Verhaar M.C. (2019). A Pro-Inflammatory Biomarker-Profile Predicts Amputation-Free Survival in Patients with Severe Limb Ischemia. Sci. Rep..

[23] Guo S., Zhang Z., Wang L., Yuan L., Bao J., Zhou J., Jing Z. (2020). Six-Month Results of Stenting of the Femoropopliteal Artery and Predictive Value of Interleukin-6: Comparison with High-Sensitivity C-Reactive Protein. Vascular.

[24] DePalma R.G., Hayes V.W., O’Leary T.J. (2021). Optimal Serum Ferritin Level Range: Iron Status Measure and Inflammatory Biomarker. Metallomics.

[25] Kremers B.M.M., Posma J.N., Heitmeier S., Glunz J., Ten Cate H., Pallares Robles A., Daemen J.H.C., Ten Cate-Hoek A.J., Mees B.M.E., Spronk H.M.H. (2022). Discovery of Four Plasmatic Biomarkers Potentially Predicting Cardiovascular Outcome in Peripheral Artery Disease. Sci. Rep..

[26] Martín-Núñez E., Pérez-Castro A., Tagua V.G., Hernández-Carballo C., Ferri C., Pérez-Delgado N., Rodríguez-Ramos S., Cerro-López P., López-Castillo Á., Delgado-Molinos A. (2022). Klotho Expression in Peripheral Blood Circulating Cells Is Associated with Vascular and Systemic Inflammation in Atherosclerotic Vascular Disease. Sci. Rep..

[27] Fatkhullina A.R., Peshkova I.O., Koltsova E.K. (2016). The Role of Cytokines in the Development of Atherosclerosis. Biochem. Mosc..

[28] Gremmel T., Perkmann T., Kopp C.W., Seidinger D., Eichelberger B., Koppensteiner R., Steiner S., Panzer S. (2015). Interleukin-6 and Asymmetric Dimethylarginine Are Associated with Platelet Activation after Percutaneous Angioplasty with Stent Implantation. PLoS ONE.

[29] Rossaint J., Margraf A., Zarbock A. (2018). Role of Platelets in Leukocyte Recruitment and Resolution of Inflammation. Front. Immunol..

[30] Tzoulaki I., Murray G.D., Lee A.J., Rumley A., Lowe G.D.O., Fowkes F.G.R. (2005). C-Reactive Protein, Interleukin-6, and Soluble Adhesion Molecules as Predictors of Progressive Peripheral Atherosclerosis in the General Population: Edinburgh Artery Study. Circulation.

[31] Apostolakis S., Vogiatzi K., Amanatidou V., Spandidos D.A. (2009). Interleukin 8 and Cardiovascular Disease. Cardiovasc. Res..

[32] Kirk G., Hickman P., McLaren M., Stonebridge P., Belch J. (1999). Interleukin-8 (IL-8) May Contribute to the Activation of Neutrophilsin Patients with Peripheral Arterial Occlusive Disease (PAOD). Eur. J. Vasc. Endovasc. Surg..

[33] Libby P. (2017). Interleukin-1 Beta as a Target for Atherosclerosis Therapy. J. Am. Coll. Cardiol..

[34] Russell K.S., Yates D.P., Kramer C.M., Feller A., Mahling P., Colin L., Clough T., Wang T., LaPerna L., Patel A. (2019). A Randomized, Placebo-Controlled Trial of Canakinumab in Patients with Peripheral Artery Disease. Vasc. Med..

[35] Bachmann M.F., Oxenius A. (2007). Interleukin 2: From Immunostimulation to Immunoregulation and Back Again. EMBO Rep..

[36] Chaparala R.P.C., Orsi N.M., Lindsey N.J., Girn R.S., Homer-Vanniasinkam S. (2009). Inflammatory Profiling of Peripheral Arterial Disease. Ann. Vasc. Surg..

[37] Mallat Z., Besnard S., Duriez M., Deleuze V., Emmanuel F., Bureau M.F., Soubrier F., Esposito B., Duez H., Fievet C. (1999). Protective Role of Interleukin-10 in Atherosclerosis. Circ. Res..

[38] Opal S.M., DePalo V.A. (2000). Anti-Inflammatory Cytokines. Chest.

[39] El Marghani A.M., Abuabaid H.M., Hurtig-Wennlöf A., Sirsjö A., Norgren L., Kjellen P. (2010). High MAPK P38 Activity and Low Level of IL-10 in Intermittent Claudication as Opposed to Stable Angina. Int. Angiol..

[40] Girn H.R.S., Orsi N.M., Homer-Vanniasinkam S. (2007). An Overview of Cytokine Interactions in Atherosclerosis and Implications for Peripheral Arterial Disease. Vasc. Med..

[41] Deser S.B., Bayoglu B., Besirli K., Cengiz M., Arapi B., Junusbekov Y., Dirican A., Arslan C. (2016). Increased IL18 MRNA Levels in Peripheral Artery Disease and Its Association with Triglyceride and LDL Cholesterol Levels: A Pilot Study. Heart Vessel..

[42] Uyemura K., Demer L.L., Castle S.C., Jullien D., Berliner J.A., Gately M.K., Warrier R.R., Pham N., Fogelman A.M., Modlin R.L. (1996). Cross-Regulatory Roles of Interleukin (IL)-12 and IL-10 in Atherosclerosis. J. Clin. Investig..

